# Increased Hospitalization and Mortality from COVID-19 in Prostate Cancer Patients

**DOI:** 10.3390/cancers13071630

**Published:** 2021-04-01

**Authors:** Dimple Chakravarty, Parita Ratnani, Stanislaw Sobotka, Dara Lundon, Peter Wiklund, Sujit S. Nair, Ashutosh K. Tewari

**Affiliations:** Department of Urology and The Tisch Cancer Institute, Icahn School of Medicine at Mount Sinai, New York, NY 10029, USA; parita.ratnani@mountsinai.org (P.R.); stanislaw.sobotka@mountsinai.org (S.S.); dara.lundon@mountsinai.org (D.L.); peter.wiklund@mountsinai.org (P.W.); sujit.nair@mountsinai.org (S.S.N.); ash.tewari@mountsinai.org (A.K.T.)

**Keywords:** COVID-19, prostate cancer, hospitalization, mortality

## Abstract

**Simple Summary:**

The COVID-19 pandemic has had a remarkable and insurmountable effect on humankind. The SARS-Co-V2 infection results in a life-threatening situation, particularly in people at risk of severe infection, including those of old age, with comorbidities, and of the male gender. Prostate cancer (PCa), the most common cancer in men, shares several risk factors with COVID-19. Our study’s aim in 286,609 patients was to assess if men with prostate cancer and SARS-Co-V2 infection have poor clinical outcomes compared with patients with non-prostate GU malignancies. We confirmed that prostate cancer patients with COVID-19 had higher hospitalization and mortality rates. Among the comorbidities reported, prostate cancer patients with diabetes had a higher likelihood of being hospitalized, while COVID-19-positive prostate cancer patients with COPD had higher mortality rates. In summary, the work presented here advocates for better management of COVID-19 in prostate cancer patients, including vaccine prioritization.

**Abstract:**

Background: Cancer patients with COVID-19 have a poor disease course. Among tumor types, prostate cancer and COVID-19 share several risk factors, and the interaction of prostate cancer and COVID-19 is purported to have an adverse outcome. Methods: This was a single-institution retrospective study on 286,609 patients who underwent the COVID-19 test at Mount Sinai Hospital system from March 2020 to December 2020. Chi-square/Fisher’s exact tests were used to summarize baseline characteristics of categorical data, and Mann–Whitney U test was used for continuous variables. Univariable logistic regression analysis to compare the hospitalization and mortality rates and the strength of association was obtained by the odds ratio and confidence interval. Results: This study aimed to compare hospitalization and mortality rates between men with COVID-19 and prostate cancer and those who were COVID-19-positive with non-prostate genitourinary malignancy or any solid cancer, and with breast cancer patients. We also compared our studies to others that reported the incidence and severity of COVID-19 in prostate cancer patients. Our studies highlight that patients with prostate cancer had higher susceptibility to COVID-19-related pathogenesis, resulting in higher mortality and hospitalization rates. Hospitalization and mortality rates were higher in prostate cancer patients with COVID-19 when compared with COVID-19 patients with non-prostate genitourinary (GU) malignancies.

## 1. Introduction

Cancer patients are at an increased risk of developing life-threatening complications from COVID-19 due to the immunosuppressive effect of the disease and of cancer-directed therapy [1,2]. COVID-19 infection and prostate cancer are significantly influenced by common comorbidities like age, diabetes, hypertension, obesity, and race, resulting in adverse outcomes [3]. Androgen, and androgen receptor signaling, a hallmark of prostate cancer, can augment COVID-19 severity due to its ability to enhance the expression of type II protease, TMPRSS2 [4,5,6,7,8,9], a central molecule that mediates severe acute respiratory syndrome coronavirus 2 (SARS-CoV-2) infection, and by dampening the innate and adaptive immune responses [3,10,11]. Several studies that have focused on evaluating the severity of COVID-19 in prostate cancer patients demonstrate that the interaction between COVID-19 and prostate cancer results in an unfavorable clinical outcome [2,12,13,14,15,16,17]. Furthermore, studies also show the anti-androgens protective effect in decreasing COVID-19 pathogenesis [15]. Due to the confluence of risk and molecular factors between prostate cancer and COVID-19, patients with prostate cancer represent a vulnerable population at an increased risk of developing severe COVID-19 infections resulting in increased hospitalization and mortality (graphical abstract). The study aimed to confirm these findings in our own Mount Sinai patient cohort (*n* = 286,609) and to evaluate if the risk of hospitalization and mortality due to COVID-19 is influenced by cancer type and specifically by prostate cancer.

## 2. Materials and Methods

### 2.1. Study Population

This was a single-institution retrospective study. De-identified data on a study cohort of (*n* = 627,465) patients were obtained from the MSHS Data Warehouse (https://msdw.mountsinai.org (accessed on 9 January 2021). A total of *n* = 286,609 patients who underwent the COVID-19 RT-PCR test at Mount Sinai Hospital system from March 2020 to December 2020 were included in this study after a waiver of informed consent documentation was approved by the MSHS Ethics Committee. COVID-19 case definition was a positive test from a real-time reverse-transcriptase PCR-based test: our institution utilized the COBAS 6800 and a laboratory developed test (LDT) using the Centers for Disease Control and Prevention (CDC) 2019-nCoV primers and probes [18]. Additional information was gathered for each patient positive for COVID-19, and this included hospitalization (yes/no), mortality (yes/no), diagnosis of cancer (PCa, solid cancer, genitourinary cancer, or breast cancer), and comorbidities.

### 2.2. Variables and Outcome

Baseline clinical data used in the analysis included age, sex, race (African American, White, Asian, Hispanic, others, and not known), smoking status (never, current, quit, and not asked), history of cancer, cancer type (prostate cancer, solid cancer, genitourinary cancer, and breast cancer), history of hypertension, diabetes, obesity, coronary artery disease, Crohn’s disease, ulcerative disease, acute respiratory disease syndrome, chronic obstructive pulmonary disease, and asthma. This study has included the following solid tumor types: melanoma, lymphoma, malignant neoplasm of cervix, colon, head, neck, face, rectum, thyroid, tongue, tonsils, bone, and brain. Descriptive statistics, including frequencies and proportions, were reported for categorical variables in the people tested for COVID-19. Descriptive statistics were performed for PCa and other genitourinary cancers such as bladder and kidney cancer for COVID-19-positive and -negative groups. This study aimed to compare hospitalization and mortality rates between men with COVID-19 and prostate cancer and COVID-19 positive with non-prostate genitourinary malignancy or any solid cancer, and with breast cancer patients.

### 2.3. Statistical Analysis

Baseline characteristics and groups were summarized using the Chi-square/Fisher’s exact tests for categorical data and Mann–Whitney U test for continuous variables. Frequencies and proportions were reported for categorical variables, while medians and interquartile ranges were reported for continuous variables. We used univariable logistic regression analysis to compare the hospitalization and mortality rates in COVID-19-positive PCa patients with COVID-19-positive patients (a) with non-prostate genitourinary malignancies, (b) with other solid tumors, and (c) with breast cancer. The strength of the association was obtained by the odds ratio (OR) and confidence interval (CI). Also, multivariable logistic regression analyses were performed to compare the impact of COVID-19 on prostate and breast cancer patients after adjusting for age. Histograms were used to summarize the results, and Forest plots were used to represent the odds ratio for hospitalization and mortality. All analysis was performed using SAS v9.4 (SAS Institute Inc., Cary, NC, USA) and Microsoft excel. All tests were two-sided, with the significance set at *p* < 0.05.

## 3. Results

### 3.1. Old Age, Gender, and Comorbidities Are Risk Factors for COVID-19

**Figure 1:**
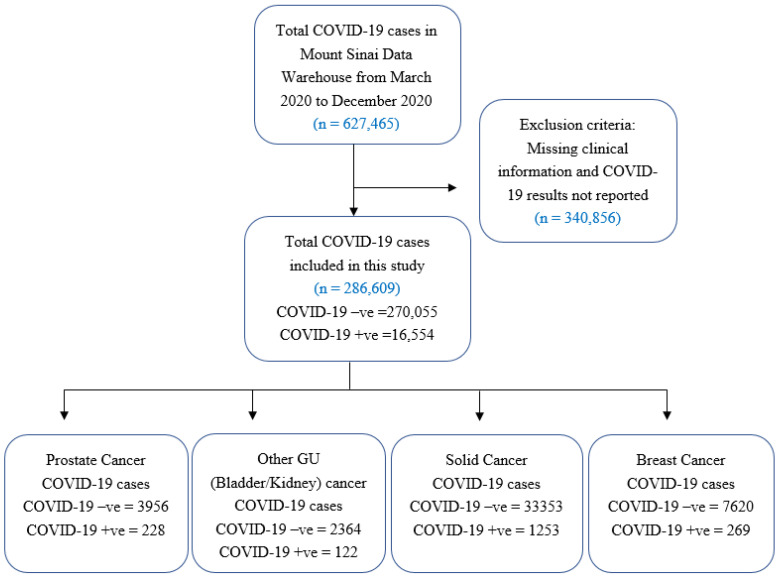
Cohort Description. A flow diagram outlines inclusion and exclusion criteria for patients selected for this single-institution retrospective study on 286,609 patients who underwent the COVID-19 test at Mount Sinai Hospital system from March 2020 to December 2020.

In Mount Sinai Health System, a total of 627,465 patients were tested for SARS-CoV-2 infection between March 2020 and December 2020. However, the results were available for 286,609 (80.18%) cases, which were included in this study. Of these, 16,554/28,6609 (5.77%) tested positive for SARS-CoV-2; 228 (1.37%) of which had a diagnosis of PCa, 122 (0.73%) had non-prostate genitourinary cancer, 1253 (7.6%) had other solid cancers, and 269 (1.62%) had breast cancer (Figure 1). Baseline characteristics for overall COVID-19 cases are summarized in Table 1, with sub-cohort analysis performed for PCa and non-prostate genitourinary cancer (bladder and kidney) cases (Tables S1 and S2).

We used the Chi-square test to calculate the baseline differences between COVID-19-positive and -negative groups for the categorical variables and Mann–Whitney U test for the continuous variables, as shown in Table 1, and *p*-values were reported. Frequencies are reported in percentages for categorical variables, and median with interquartile range for continuous variables. We found a significant difference in age, race, sex, smoking status, cancer type, and comorbidities such as obesity, hypertension, coronary artery disease, diabetes, Crohn’s disease, ulcerative colitis, and asthma between the COVID-19-positive and -negative groups with a *p*-value of <0.05 (Table 1). Further, in our analysis of PCa and GU cancer patients, we observed that older patients (≥61 years) had a higher likelihood of COVID-19 positivity (Tables S1 and S2). We also observed a higher incidence of comorbidities such as obesity, hypertension, coronary artery disease, diabetes, and asthma in GU cancer patients (*p* < 0.05) who tested positive in this cohort (Table S2).

We studied hospitalization and mortality rates in prostate cancer patients who tested positive for COVID-19 (*n* = 228; Tables S3 and S4). Significantly higher hospitalization (*p* = 0.0016) and mortality (*p* = 0.0003) rates were observed in the older patients (≥69 years). The median age was 74 (69, 82) for patients hospitalized and was 77 (73, 89) for the patients that died.

### 3.2. Prostate Cancer Patients Had a Higher Likelihood of Severe Illness Due to COVID-19

**Figure 2:**
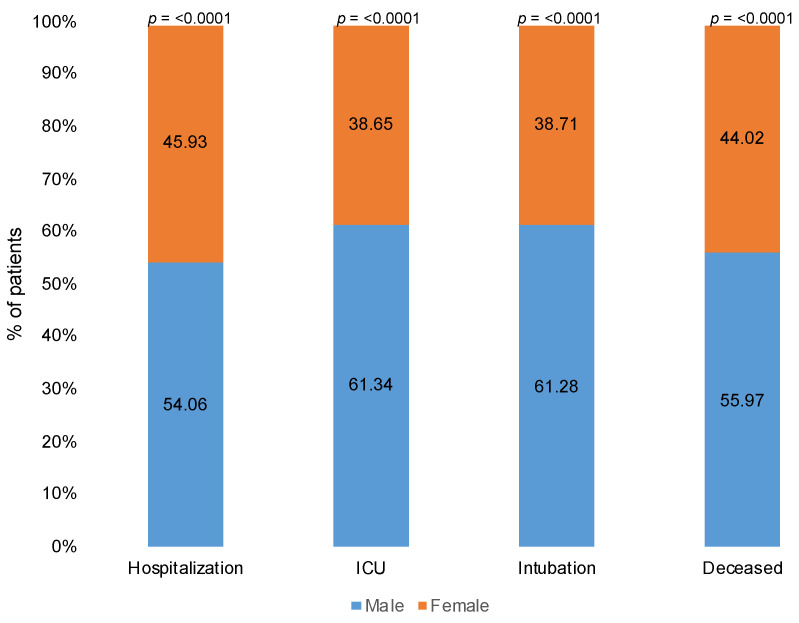
Hospitalization, ICU, intubation, and mortality in patients with SARS-CoV-2 by sex. Stacked column graph demonstrates that COVID-19-positive male patients developed more severe disease compared with females, with significant difference in rates of hospitalization, ICU admits, intubation, and mortality. Frequencies are reported in percentages for categorical variables, and *p*-values were computed using Chi-square test.

**Figure 3:**
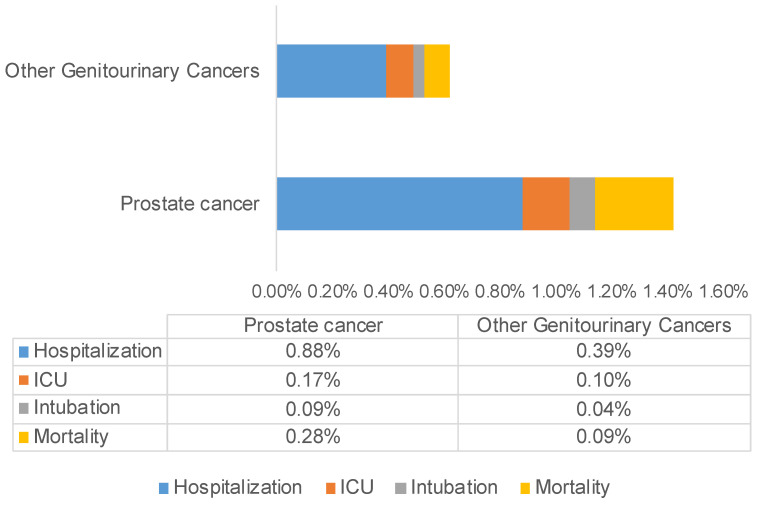
Comparison of outcomes in COVID-19-positive prostate cancer vs. non-prostate GU cancer (bladder and kidney) patients. Data presented in the clustered bar chart show higher rate of hospitalization, ICU admits, intubation, and mortality in prostate cancer patients with COVID-19.

Amongst the comorbidities reported, prostate cancer patients with diabetes had a higher likelihood of being hospitalized (*p* = 0.0054), while COVID-19-positive prostate cancer patients with COPD had higher mortality (*p* = 0.0043). COVID-19-positive male patients developed more severe disease compared with females, with a significant difference in rates of hospitalization (54.06%, *p* < 0.0001), intensive care unit (ICU) admits (61.34%, *p* < 0.0001), intubation (61.28%, *p* < 0.0001), and mortality (55.97%, *p* < 0.0001) (Figure 2). Furthermore, a comparison between the COVID-19-positive prostate cancer patients and other patients with non-prostate GU malignancies (kidney and bladder cancer) revealed a higher percentage of hospitalization (64.91% vs. 56.33%), ICU admits (12.71% vs. 11.97%), intubation (7.02% vs. 4.92%), and mortality (21.05% vs. 17.6%) in PCa patients with COVID-19. In Figure 3, we represent the percentages of hospitalization, ICU admits, intubation, and mortality in COVID-19-positive prostate cancer and other genitourinary (bladder and kidney) cancer patients from the total COVID-19-positive population in our cohort.

### 3.3. Prostate Cancer Patients Reported Higher Hospitalization and Mortality Rates Due to COVID-19

We further compared the hospitalization and mortality rates in COVID-19-positive prostate cancer patients with COVID-19-positive patients with a history of solid tumors. The overall hospitalization (64.91% vs. 47.34%; *p*-value ≤ 0.0001) and mortality (21.05% vs. 15.85%; *p*-value = 0.0547) rates were higher in PCa patients with COVID-19 compared with COVID-19 patients with solid cancers (Table 2).

**Figure 4:**
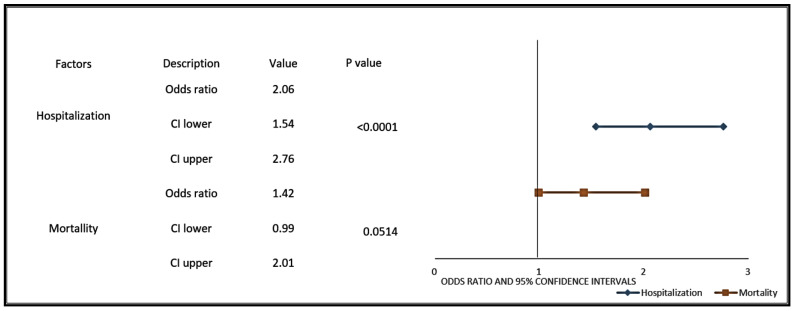
Prostate cancer patients with COVID-19 have a higher likelihood of hospitalization and mortality when compared to COVID-19-positive patients with other solid tumors. Forest plots represent odds ratios for hospitalization and mortality in COVID-19-positive prostate cancer patients compared to patients with other solid tumors. The line represents the lower and upper limits of the 95% confidence intervals. Lower OR values favor decreased risk for hospitalization and mortality, whereas higher values indicate increased risk for hospitalization and mortality from COVID-19.

The COVID-19-positive prostate cancer patients were more likely to be hospitalized (OR: 2.06, 95% CI 1.54–2.76; *p*-value ≤ 0.0001) or die (OR: 1.42, 95% CI 0.99–2.01; *p*-value = 0.0514) from COVID-19 than COVID-19-positive patients with a history of solid cancer (Figure 4). A subgroup analysis comparing COVID-19-positive PCa patients with COVID-19-positive males with solid tumors showed higher hospitalization rates, ICU, and mortality due to COVID-19 in the PCa patients (*p*-value < 0.0001, Table S5). Several studies have discussed racial disparities in incidence and outcomes of COVID-19, with African American men having the highest rate of incidence and severity [2]. Our data independently corroborate these findings and demonstrate a higher incidence of COVID-19 among African American men. A significant difference in the distribution of COVID-19-positive PCa patients and other solid cancer patients is seen for ethnicity, with African American men (5.8% vs. 3.9%; *p*-value = 0.003), White men (3.7% vs. 2.82%; *p*-value = 0.0412), Asian men (8.33% vs. 2.20%; *p*-value = 0.0033), and Hispanic men (8.46% vs. 5.37%, *p*-value = 0.002; Table S6) having a higher incidence of prostate cancer. Frequency of hospitalization was significantly higher in the African American (31.75% vs. 26.73%, *p*-value = 0.0045) and Hispanic (29.05% vs. 26.42%; *p*-value = 0.0003) prostate cancer patients compared with patients with solid cancers (Table 3).

However, on further analysis, we found the mortality rate was higher in the COVID-19-positive White population in PCa vs. solid cancer patients (41.67% vs. 27.52%, *p*-value = 0.0014). The hospitalization (64.91% vs. 56.33%, *p* value = 0.0479) and mortality rates (21.05% vs. 17.6%; *p*-value = 0.0421) were also higher with minimal significance in COVID-19-positive prostate cancer patients when compared with other COVID-19-positive patients with non-prostate genitourinary malignancies (bladder and kidney) (Table 4).

**Figure 5:**
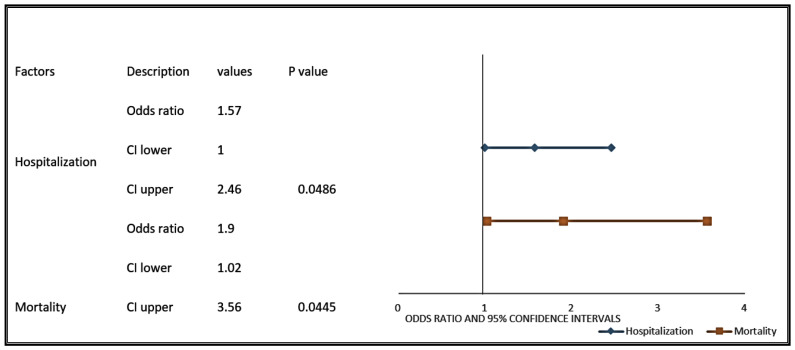
Prostate cancer patients with COVID-19 have a higher likelihood of hospitalization and mortality when compared with COVID-19-positive patients with other genitourinary cancers. Forest plots were used to represent the odds ratios for hospitalization and mortality in COVID-19-positive prostate cancer patients compared to patients with other genitourinary cancers. The line represents the lower and upper limits of the 95% confidence intervals. Lower OR values favor decreased risk for hospitalization and mortality, whereas higher values indicate increased risk for hospitalization and mortality from COVID-19.

The COVID-19-positive prostate cancer patients were more likely to be hospitalized (OR: 1.57; 95% CI 1–2.46, *p*-value = 0.0486) or die (OR: 1.9; 95% CI 1.02–3.56; *p*-value = 0.0445) from COVID-19 when compared with non-prostate GU cancer (bladder and kidney) patients (Figure 5).

### 3.4. Prostate Cancer Patients Had Higher Hospitalization and Mortality Rates Compared with Breast Cancer Patients Due to COVID-19

Both prostate and breast cancer are regulated by hormones androgen and estrogen, respectively, and share oncogenic regulatory pathways [19]. Nuclear receptor signaling is a hallmark of both cancer types, albeit regulated by two different nuclear receptor proteins. Androgens and Androgen receptor (AR) play a critical role in prostate cancer progression, while estrogen receptor deregulation is associated with breast cancer. Interestingly the immunosuppressive effects of androgens in the context of virus infection are well studied [3,10,11,20]. We compared hospitalization and mortality rates in prostate and breast cancer patients with COVID-19. When compared to breast cancer patients with COVID-19, we observed higher rates of hospitalization (64.91% vs. 42.75%; *p*-value ≤ 0.0001), ICU (12.71% vs. 4.83%; *p*-value = 0.0016), intubation (7.01% vs. 2.97%; *p*-value = 0.0361), and mortality (21.05% vs. 10.78%; *p*-value = 0.0016) in COVID-19-positive PCa patients (Table 5).

**Figure 6:**
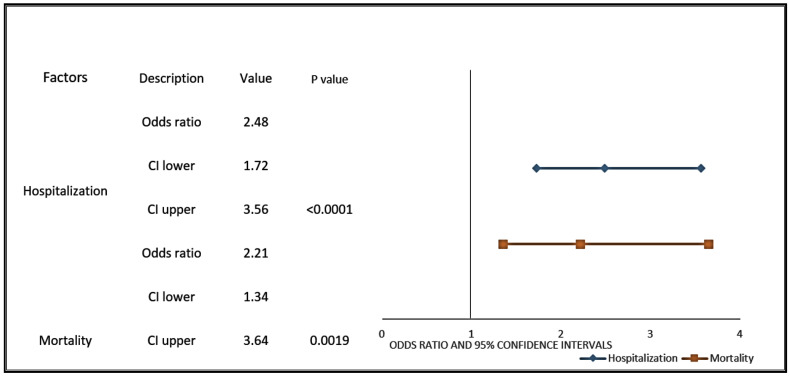
Prostate cancer patients with COVID-19 have a higher likelihood of hospitalization and mortality when compared with COVID-19-positive patients with breast cancer. Forest plots were used to represent the odds ratios for hospitalization and mortality in COVID-19-positive prostate cancer patients compared with COVID-19-positive patients with breast cancer. The line represents the lower and upper limits of the 95% confidence intervals. Lower OR values favor decreased risk for hospitalization and mortality, whereas higher values indicate increased risk for hospitalization and mortality from COVID-19.

Figure 6 shows the unadjusted odds ratio of hospitalization (OR: 2.48, 95% CI 1.72–3.56; *p*-value ≤ 0.0001) and mortality (OR: 2.21, 95% CI 1.34–3.64; *p*-value = 0.0019) in forest plot. Since these two cancer types occur in different age groups, we performed a multivariable analysis where we partially adjusted the odds ratio: age was adjusted for hospitalization and mortality. The differences in hospitalization (Adjusted Odds Ratio (aOR): 1.63, 95% CI 1.09–2.43; *p*-value = 0.0171) and age (aOR: 1.04, 95% CI 1.03–1.06; *p*-value ≤ 0.0001) were found significantly different in COVID-19-positive PCa patients compared with breast cancer patients (Table S7). Similarly, after adjusting for age, higher mortality was seen in COVID-19-positive prostate cancer patients compared with breast cancer patients (Table S8).

### 3.5. Prostate Cancer Patients Reported Higher Incidence, Hospitalization, and Mortality Rates Due to COVID-19 Across Several Cohorts

**Figure 7:**
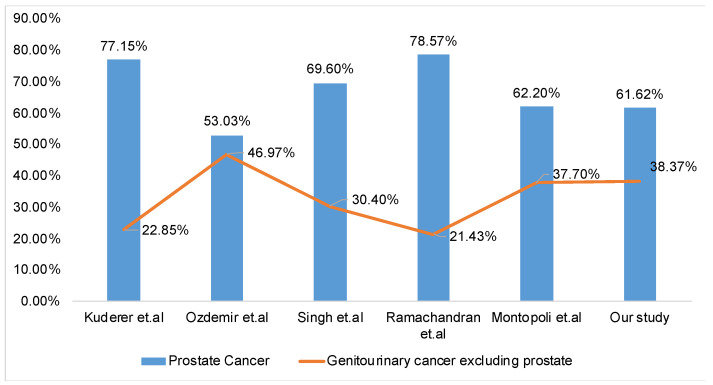
Comparison of the incidence of COVID-19 across different studies in Prostate Cancer vs. non-prostate genitourinary cancer (bladder and kidney) patients. A clustered column chart with a line graph compares the incidence of COVID-19 across different studies in prostate cancer vs. non-prostate genitourinary cancer (bladder and kidney) patients. A higher incidence of COVID-19 was reported in prostate cancer patients across several studies.

**Figure 8:**
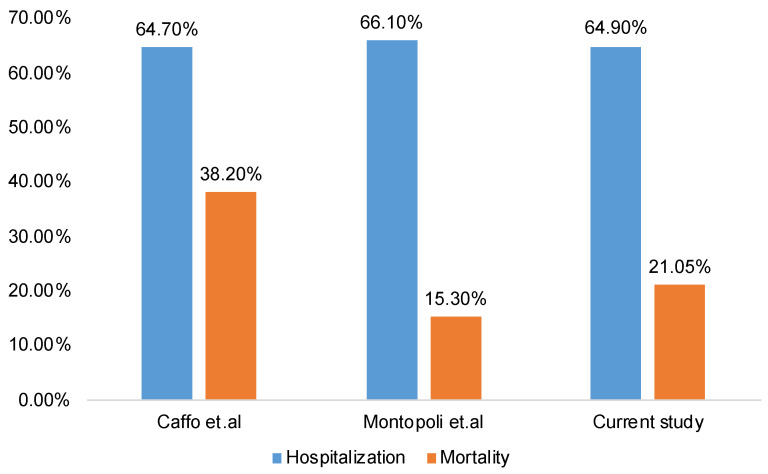
Incidence of hospitalization and mortality in COVID-19-positive prostate cancer patients across different studies. Clustered column chart demonstrates a higher incidence of hospitalization and mortality in COVID-19-positive prostate cancer patients across different studies, including this study.

We performed a systematic review of studies on COVID-19 in prostate cancer patients. A PubMed search of “prostate cancer” and “COVID-19” resulted in nine studies [2,12,13,14,15,16,17,21,22]. After reviewing the nine publications, which did include relevant information on the incidence of COVID-19 in prostate cancer patients, we excluded four studies [2,12,13,16] that did not include details of the reference group in their analysis. The five studies that were selected reported the incidence rate of COVID-19 in patients with prostate and other genitourinary cancer (Figure 7). In all these studies, including our study, prostate cancer incidence was higher than the incidence of non-prostate genitourinary cancers. Of these five studies, only two studies provided information on the hospitalization and mortality rates in COVID-19-positive PCa patients and were used for analysis (Figure 8). These two studies reported a higher incidence of hospitalization and mortality in COVID-19-positive PCa patients, similar to our study [12,15] (Figure 8). Studies used for comparisons, presented in Figure 7 and Figure 8, include data from the Turkish National Registry Data [21], COVID-19 and Cancer Consortium (CCC19) Database [14], Detroit study [17], a single-center retrospective study from New York City [22], and data from Veneto hospitals in Italy [15].

## 4. Discussion

Interaction between the SARS-CO-V-2 virus and the host factors underscores the extent of COVID-19 pathogenesis and severity. Cancer is a risk factor for severe COVID-19, and the effects are more pronounced in cancer patients receiving anticancer treatments with immunosuppressive effects. Interestingly the impact of COVID-19 on cancer is also dependent on cancer type [2]. Our study establishes that prostate cancer patients are more susceptible to adverse effects of COVID-19 when compared with patients with other GU malignancies. Based on these studies, we surmise that the severity of infection observed in prostate cancer may be attributed to the unfavorable interaction between virus factors and prostate cancer. Work from us and others has shown that old age, comorbidities such as diabetes and hypertension, behavioral factors like smoking, and molecular factors like androgen receptor signaling and TMPRSS2 may contribute to the increased risk of COVID-19 for prostate cancer patients [3].

Several studies have previously discussed the critical role of TMPRSS2 in the context of SARS-Co-V-2 infection, including a recent review by our team wherein we have discussed the association between TMPRSS2, AR, and SARS-Co-V2 in the context of prostate cancer [3]. Several other studies have explored the mechanism of TMPRSS2 regulation by AR and implications for COVID-19. The androgen receptor is an essential signaling regulator of prostate cellular and physiological function. In conjunction with the androgen receptor, androgens activate TMPRSS2 expression, which plays a vital role in internalizing the SARS-CO-V2 virus and productive infection. Thus, TMPRSS2 was logically pursued as a potential therapeutic target to mitigate SARS-Co-V2 infection. Camostat mesylate, a serine protease inhibitor, was found to block SARS-CoV-2 infection in the lungs by interfering with the activity of TMPRSS2 [23]. The regulation of TMPRSS2 by AR is also seen in the lungs [24] and has significant implications for COVID-19. A recent study demonstrated that blocking androgen receptors using AR antagonists, e.g., enzalutamide, apalutamide or AR degrader ARD-61, Bromodomain and extra-terminal domain (BET) agonist, or BET degrader, has a potential to block against SARS-Co-V2 infection [25]. Another study showed that a combination of antiviral drugs with TMPRSS2 or AR inhibitors could prevent SARS-Co-V2 infection [24].

Along these lines, androgen deprivation therapy was partially shown to protect prostate cancer patients from COVID-19 [15]. Androgens play distinct roles in immune cells’ functioning and affect innate and adaptive immune responses [3,10,26]. Several studies using human and preclinical animal models have attempted to delineate further the mechanisms by which androgens exert these effects on the immune system. In a macaque model, androgens have been shown to alter the viral load [27]. During lymphocytic choriomeningitis virus (LCMV) infection, androgens have been shown to block antigen-specific T cell responses [11]. The extent of immune responses to viruses and vaccines can be modulated by testosterone (androgen), and inhibition of T-helper 1 differentiation has been postulated as a mechanism underlying the immunosuppressive effect of androgens on the immune response [28]. Several other studies support this view. A study using a systems biology approach demonstrated that androgens influence lipid metabolism genes to impact immune response in response to influenza vaccination [29]. Androgen receptor has been shown to promote hepatitis B virus-induced liver cancer and facilitate Kaposi’s sarcoma-associated herpesvirus (KSHV) infection [30,31]. Compared with females, males are more susceptible to viral, bacterial, fungal, and parasite infections, and androgens have been postulated to play a significant role in modulating the immunocompetence [31,32,33].

COVID-19 severity is associated with cytokine storm and elevated levels of pro-inflammatory cytokines in peripheral blood [3,34,35]. Several COVID-19-induced pro-inflammatory cytokines can augment prostate cancer [3]. We posit that the effects of androgens and androgen receptor signaling on TMPRSS2 and immune response could potentially contribute to the increased severity of COVID-19 in prostate cancer patients. Androgens and AR can enhance TMPRSS2 expression, which is critical for virus entry, while also suppressing the innate and adaptive immune responses [3]. Suppressive effects of androgens and AR can facilitate a potent viral infection and a cytokine storm. The increased hospitalization and mortality in prostate cancer patients due to COVID-19 could result from the aforementioned risk factors shared between both diseases.

Strengths: This study demonstrates that prostate cancer patients with COVID-19 had a poor outcome associated with higher hospitalization and mortality. Interestingly, the hospitalization rates were higher when compared with patients with non-prostate genitourinary cancer (bladder and kidney) and other solid cancers. In addition to the hospitalization and mortality rates, the intubation and ICU rates resulting from COVID-19 were higher in patients with prostate cancer when compared with breast cancer patients. Further, the frequency of hospitalization was significantly higher in African Americans and Hispanics (prostate cancer patients) than in patients with solid cancers. This study provides a compelling rationale to implement effective measures for clinical management of prostate cancer patients during the pandemic, including early COVID-19 vaccination. The success of strategies for better clinical management of COVID-19 in prostate cancer patients will likely involve collaboration between urologists and infectious disease physicians.

Limitations: Our study does not include the treatment history, such as chemotherapy or androgen receptor axis-targeted therapies, so we could not study the association or impact of that on COVID-19-positive prostate cancer patients. Multivariable analysis to determine the impact of other clinical covariates such as demographics, behavioral factors, and comorbidities on COVID-19 and/or prostate cancer outcome could not be performed due to the relatively small study cohort; COVID-19-positive prostate cancer patients (*n* = 228). Additionally, survival analysis was not feasible due to a lack of long-term follow-up and is a part of an active investigation in our laboratory.

While the COBAS SARS-CoV-2 test was designed to minimize the likelihood of false-positive results, in the absence of a gold standard, determining a false-positive rate could not be calculated by the assay developers. Previously published work has identified that the LDT at our institution has indicated excellent agreement with the COBAS SARS-CoV-2 test [18]. The FDA has recently (12 March 2021) stated that the COBAS test might result in false-positive results, which the developer states may be related to assay tube leaks or abnormal PCR cycling in the reaction tubes. This is certainly a limitation of this or any study that uses such PCR-based tests [36].

## 5. Conclusions

A single-institute retrospective study presented in this manuscript demonstrates that patients diagnosed with prostate cancer are at significantly higher risk of COVID-19 severity and related death. Hospitalization and mortality rates were higher in prostate cancer patients with COVID-19 than in COVID-19 patients with non-prostate GU malignancies, concordant to those reported in other studies. Further studies are required to elucidate the underlying mechanisms contributing to the increased risk of disease severity in prostate cancer patients. There is a need to identify those at most significant risk and those in whom targeted therapies and vaccination offer the most significant therapeutic benefit. Further studies are also warranted to understand how COVID-19 would impact prostate cancer.

## Figures and Tables

**Table 1:** Demographic and clinical characteristics of all patients tested for COVID-19.

Covariates	COVID − ve (*n* = 270,055)	COVID + ve (*n* = 16,554)	*p*-Value
Median Age (IQR):	53 (35, 67)	59 (41, 73)	<0.0001
SEX			<0.0001
Male	121,989 (45.17%)	8476 (51.2%)	
Female	148,066 (54.83%)	8078 (48.80%)	
Race/Ethnicity:			<0.0001
African American	53,155 (19.68%)	3763 (22.73%)	
White	96,989 (35.91%)	4543 (27.44%)	
Asian	18,741 (6.94%)	978 (5.91%)	
Hispanic	55,592 (20.59%)	4307 (26.02%)	
Others	25,363 (9.39%)	2195 (13.26%)	
Unknown	20,215 (7.49%)	768 (4.64%)	
Smoking:			<0.0001
Current	29,126 (10.79%)	1006 (6.08%)	
Never	171,456 (63.49%)	11,196 (67.63%)	
Not Asked	6271 (2.32%)	697 (4.21%)	
Quit	63,202 (23.40%)	3655 (22.08%)	
Cancer Type:			<0.0001
Other Cancer	37,680 (13.95%)	1524 (9.21%)	
Prostate Cancer	3956 (1.46%)	228 (1.38%)	
No cancer	228,419 (84.58%)	14,802 (89.42%)	
Genitourinary cancer	6320 (15.93%)	350 (2.11%)	
Solid cancer	33,353 (84.07%)	1253 (7.56%)	
Hypertension:			<0.0001
No	203,152 (75.23%)	11,492 (69.42%)	
yes	66,903 (24.77%)	5062 (30.58%)	
Diabetes:			<0.0001
No	235,991 (87.39%)	13,499 (81.55%)	
Yes	34,064 (12.61%)	3055 (18.45%)	
Coronary Artery Disease:			<0.0001
No	243,582 (90.20%)	14,623 (88.34%)	
Yes	26,473 (9.8%)	1931 (11.66%)	
Crohns Disease			<0.0001
No	267,607 (99.09%)	16,493 (99.63%)	
Yes	2448 (0.91%)	61 (0.37%)	
Ulcerative Colitis			<0.0001
No	267,943 (99.22%)	16,473 (99.51%)	
Yes	2112(0.78%)	81 (0.49%)	
Acute Respiratory Distress Syndrome			<0.0001
No	270,000 (99.98%)	16,516 (99.77%)	
Yes	55 (0.02%)	38 (0.23%)	
Chronic Obstructive Pulmonary Disease:			0.4716
No	261,313 (96.76%)	16,035 (96.86%)	
Yes	8742 (3.24%)	519 (3.14%)	
Obesity:			<0.0001
No	250,001 (92.57%)	15,163 (91.6%)	
Yes	20,054 (7.43%)	1391 (8.4%)	
Asthma:			<0.0001
No	251,098 (92.98%)	15,569 (94.05%)	
Yes	18,957 (7.02%)	985 (5.95%)	

**Table 2:** Frequency distribution of COVID-19-positive prostate cancer patients vs. patients with solid cancer.

Factors	Prostate Cancer	Solid Cancer	*p*-Value	Total COVID-19-Positive Cases
Hospitalization	148 (64.91%)	651 (47.34%)	<0.0001	7729
ICU	29 (12.71%)	138 (10.03%)	0.2194	1684
Intubation	16 (7.01%)	79 (5.74%)	0.4512	1121
Death	48 (21.05%)	218 (15.85%)	0.0547	1990
Total	228	1375		

**Table 3:** Hospitalization rate by race in COVID-19-positive prostate cancer compared with COVID-19-positive patients with solid cancer.

Factors	Prostate Cancer	Solid Cancer	*p*-Value
African American	47 (31.75%)	174 (26.73%)	0.0045
White	38 (25.67%)	218 (33.49%)	0.4665
Asian	3 (0.20%)	27 (4.15%)	1.00
Hispanic	43 (29.05%)	172 (26.42%)	0.0003
Others	15 (10.13%)	53 (8.14%)	0.0106
Unknown	2 (1.35%)	7 (1.08%)	0.5658
Total	148	651	

**Table 4:** Frequency distribution of outcomes in COVID-19-positive prostate vs. non-prostate GU cancer patients.

Factors	Prostate Cancer	Non-Prostate Genitourinary Cancer	*p*-Value
Hospitalization	148 (64.91%)	66 (56.33%)	0.0479
ICU	29 (12.71%)	17 (11.97%)	0.7485
Intubation	16 (7.01%)	7 (4.92%)	0.6452
Death	48 (21.05%)	15 (17.6%)	0.0421

**Table 5:** Frequency distribution of outcomes in COVID-19-positive prostate. vs. breast cancer patients.

Factors	Prostate Cancer	Breast Cancer	*p*-Value
Hospitalization	148 (64.91%)	115 (42.75%)	<0.0001
ICU	29 (12.71%)	13 (4.83%)	0.0016
Intubation	16 (7.01%)	8 (2.97%)	0.0361
Death	48 (21.05%)	29 (10.78%)	0.0016

## Data Availability

The data presented in this study are available on request. The systematic review was done on data from publications listed in References [14,15,17,21,22].

## Supplementary Materials

The following are available online at https://www.mdpi.com/article/10.3390/cancers13071630/s1, Table S1: Baseline characteristics of COVID-19 − ve and COVID-19 + ve prostate cancer patients, Table S2: Baseline characteristics of COVID-19 − ve and COVID-19 + ve Genitourinary cancer patients, Table S3: Baseline Characteristics for hospitalization in COVID-19 positive Prostate. cancer patients, Table S4: Baseline Characteristics for mortality in COVID-19 positive Prostate cancer patients, Table S5: Frequency distribution in COVID-19 positive Prostate vs solid tumor (male) patients, Table S6: Incidence of COVID-19 by Race in prostate cancer patients compared to patients with solid cancer, Table S7: Adjusted Odds Ratio showing hospitalization rates in COVID-19 positive prostate cancer patients compared to breast cancer patients, Table S8: Adjusted Odds Ratio showing mortality rates in COVID-19 positive prostate cancer patients compared to breast cancer patients.
