# Supplementary material for: Increased Hospitalization and Mortality from COVID-19 in Prostate Cancer Patients
Source: Cancers (Basel). 2021 Apr 1;13(7):1630. doi: 10.3390/cancers13071630 (PMC8037308; doi:10.3390/cancers13071630)
Supplement: Supplementary file 1 [file cancers-13-01630-s001.pdf]

Supplementary Materials: Increased Hospitalization and Mortality from COVID-19 in Prostate Cancer Patients

Dimple Chakravarty, Parita Ratnani, Stanislaw Sobotka, Dara Lundon, Peter Wiklund, Sujit. S. Nair and Ashutosh K. Tewari

Table S1. Baseline characteristics of COVID-19 − ve and COVID-19 + ve prostate cancer patients.

Covariates	COVID-19 – ve (*n* = 3956)	COVID-19 + ve (*n* = 228)	*p*-Value
Median Age [IQR]:	71 (64, 78)	73 (67, 79)	0.0055
Race/Ethnicity:			<0.0001
African American	1054 (26.64%)	64 (28.07%)	
White	1697 (42.90%)	64 (28.07%)	
Asian	77 (1.95%)	7 (3.07%)	
Hispanic	761 (19.24%)	70 (30.70%)	
Others	285 (7.20%)	20 (8.77%)	
Unknown	82 (2.07%)	3 (1.32%)	
Smoking:			0.3195
Current	314 (7.94%)	13 (5.70%)	
Never	1769 (4472%)	106 (46.49%)	
Not Asked	33 (0.83%)	0	
Quit	1840 (46.51%)	109 (47.81%)	
HTN:			0.2153
No	1867 (47.19%)	98 (42.98%)	
Yes	2089 (52.81%)	130 (57.02%)	
Diabetes:			0.0003
No	3151 (79.65%)	159 (69.74%)	
Yes	805 (20.35%)	69 (30.26%)	
Asthma			0.8638
No	3755 (94.92%)	217 (95.18%)	
Yes	201 (5.08%)	11 (4.82%)	
COPD			0.1024
No	3744 (94.64%)	210 (92.11%)	
Yes	212 (5.36%)	18 (7.89%)	
Crohn's Disease			1
No	3938 (99.54%)	227 (99.56%)	
Yes	18 (0.46%)	1 (0.44%)	
Ulcerative_colitis			0.1526
No	3934 (99.44%)	225 (98.68%)	
Yes	22 (0.56%)	3 (1.32%)	
Obesity			0.0012
No	3705 (93.66%)	201 (88.16%)	
Yes	251 (6.34%)	27 (11.84%)	
Coronary Artery Disease			0.6439
No	3037 (76.77%)	172 (75.44%)	
Yes	919 (23.23%)	56 (24.56%)	

Table S2. Baseline characteristics of COVID-19 − ve and COVID-19 + ve Genitourinary cancer patients.

Covariates	COVID-19 – ve (*n* = 6320)	COVID-19 + ve (*n* = 350)	p-Value
Median Age [IQR]:	69 (61, 77)	71 (63, 79)	0.0343

Race/Ethnicity:			0.0023
African American	458 (19.37%)	30 (24.59%)	
White	1149 (48.60%)	41 (33.61%)	
Asian	104 (4.40%)	3 (2.46%)	
Hispanic	468 (19.8%)	34 (27.87%)	
Others	148 (6.26%)	14 (11.48%)	
Unknown	37 (1.57%)	0	
Smoking:			0.0016
Current	309 (13.07%)	3 (2.46%)	
Never	856 (36.21%)	59 (48.36%)	
Not Asked	32 (1.35%)	2 (1.64%)	
Quit	1167 (49.37%)	58 (47.54%)	
HTN:			0.0381
No	1235 (52.24%)	52 (42.62%)	
Yes	1129 (47.76%)	70 (57.38%)	
Diabetes:			0.0073
No	1902 (80.46%)	86 (70.49%)	
Yes	462 (19.54%)	36 (29.51%)	
Asthma			0.0059
No	2234 (94.50%)	108 (88.52%)	
Yes	130 (5.50%)	14 (11.48%)	
COPD			0.8090
No	2203 (93.19%)	113 (92.62%)	
Yes	161 (6.81%)	9 (7.38%)	
Crohn's Disease			1
No	2351 (99.45%)	122 (100%)	
Yes	13 (0.55%)	0	
Ulcerative_colitis			1
No	2344 (99.15%)	121 (99.18%)	
Yes	20 (0.85%)	1 (0.82%)	
Obesity			<0.0001
No	2197 (92.94%)	101 (82.79%)	
Yes	167 (7.06%)	21 (17.21%)	
Coronary Artery Disease			0.0005
No	1988 (84.09%)	88 (72.13%)	
Yes	376 (15.91%)	34 (27.87%)	

Table S3. Baseline Characteristics for hospitalization in COVID-19 positive Prostate. cancer patients.

**Covariates**	**Hospitalization – ve (*n* = 80)**	**Hospitalization +ve (*n* = 148)**	**p-Value**
Median Age [IQR]:	70 (64, 77)	74 (69, 82)	0.0016
Race/Ethnicity:			0.3125
African American	17 (21.25%)	47 (31.76%)	
White	26 (32.5%)	38 (25.68%)	
Asian	4 (5%)	3 (2.03%)	
Hispanic	27 (33.75%)	43 (29.05%)	
Others	5 (6.25%)	15 (10.14%)	
Unknown	1 (1.25%)	2 (1.35%)	
Smoking:			0.2923
Current	7 (8.75%)	6 (4.05%)	
Never	38 (47.5%)	68 (45.95%)	
Not Asked			
Quit	35 (43.75%)	74 (50%)	
HTN:			0.3110

No	38 (47.5%)	60 (40.54%)	
yes	42 (52.5%)	88 (59.46%)	
Diabetes:			0.0054
No	65 (81.25%)	94 (63.51%)	
Yes	15 (18.75%)	54 (36.49%)	
CAD:			0.1339
No	65 (81.25%)	107 (72.3%)	
Yes	15 (18.75%)	41 (27.7%)	
Crohns Disease			1
No	80 (100)	147 (99.32%)	
Yes	0	1 (0.68%)	
Ulcerative Colitis			1
No	79 (98.75%)	146 (98.65%)	
Yes	1 (1.25%)	2 (1.35%)	
ARDS			
No	80 (100%)	148 (100%)	
COPD:			0.2334
No	76 (95%)	134 (90.54%)	
Yes	4 (5%)	14 (9.46%)	
Obesity:			0.5268
No	72 (90%)	129 (87.16%)	
Yes	8 (10%)	19 (12.84%)	
Asthma:			0.2
No	74 (92.5%)	143 (96.62%)	
Yes	6 (7.5%)	5 (3.38%)	

Table S4. Baseline Characteristics for mortality in COVID-19 positive Prostate cancer patients.

**Covariates**	**Deceased –ve (*n* = 180)**	**Deceased + ve (*n* = 48)**	***p*-Value**
Median Age [IQR]:	71 (67, 79)	77.5 (73, 89)	0.0003
Race/Ethnicity:			0.0114
African American	52 (28.89%)	12 (25%)	
White	44 (24.44%)	20 (41.67%)	
Asian	5 (2.78%)	2 (4.17%)	
Hispanic	63 (35%)	7 (14.58%)	
Others	15 (8.33%)	5 (10.42%)	
Unknown	1 (0.56%)	2 (4.17%)	
Smoking:			0.2687
Current	10 (5.56%)	3 (6.25%)	
Never	79 (43.89%)	27(56.25%)	
Not Asked			
Quit			
HTN:			0.5924
No	79 (43.89%)	19 (39.58%)	
yes	101 (56.11%)	29 (60.42%)	
Diabetes:			0.3717
No	123 (68.33%)	36 (75%)	
Yes	57 (31.67%)	12 (25%)	
CAD:			0.2257
No	139 (77.22%)	33 (68.75%)	
Yes	41 (22.78%)	15 (31.25%)	
Crohns Disease			1
No	179 (99.44%)	48 (100%)	
Yes	1 (0.56%)	0	

Ulcerative Colitis			1
No	177 (98.33%)	48 (100%)	
Yes	3 (1.67%)	0	
ARDS			
No	180 (100%)	48 (100%)	
COPD:			0.0043
No	171 (95%)	39 (81.25%)	
Yes	9 (5%)	9 (18.75%)	
Obesity:			0.3971
No	157 (87.22%)	44 (91.67%)	
Yes	23 (12.78%)	4 (8.33%)	
Asthma:			1
No	171 (95%)	46 (95.83%)	
Yes	9 (5%)	2 (4.17%)	

Table S5. Frequency distribution in COVID-19 positive Prostate vs solid tumor (male) patients.

Factors	Prostate Cancer	Solid Cancer (male)	*p*-Value	Total COVID-19 positive cases
Hospitalization	148 (64.91%)	317 (49.45%)	<0.0001	7729
ICU	29 (12.71%)	74(11.54%)	<0.0001	1684
Intubation	16 (7.01%)	53 (8.26%)	0.4511	1121
Death	48 (21.05%)	117 (18.25%)	0.00023	1990
Total	228	641		

Table S6. Incidence of COVID-19 by Race in prostate cancer patients compared to patients with solid cancer.

Factors	Prostate Cancer	Solid Cancer	P value
African American	64 (5.80%)	321 (3.90%)	0.003
White	64 (3.70%)	400 (2.82%)	0.0412
Asian	7 (8.33%)	73 (2.20%)	0.0033
Hispanic	70 (8.46%)	444 (5.37%)	0.0002
Others	20 (6.55%)	120 (4.56%)	0.1226
Unknown	3 (3.52%)	17 (3.31%)	1.00
Total	4133	37078	

Table S7. Adjusted Odds Ratio showing hospitalization rates in COVID-19 positive prostate cancer patients compared to breast cancer patients.

Factors	Odds Ratio (95% CI)	p-Value
Age	1.04 (1.03, 1.06)	<0.0001
Hospitalization		
Absent	Ref.	
Present	1.63 (1.09, 2.43)	0.0171

Table S8. Adjusted Odds Ratio showing mortality rates in COVID-19 positive prostate cancer patients compared to breast cancer patients.

Factors	Odds Ratio (95% CI)	p-Value
Age	1.04 (1.03, 1.06)	<0.0001
Mortality		
Absent	Ref.	
Present	2.04 (1.20, 3.46)	0.0082
